# Potential predisposing features of external cervical resorption: An observational study

**DOI:** 10.1111/iej.14166

**Published:** 2024-11-19

**Authors:** Shanon Patel, Francesc Abella, Kreena Patel, AbdulAziz Bakhsh, Paul Lambrechts, Nassr Al‐Nuaimi

**Affiliations:** ^1^ Faculty of Dentistry, Oral & Craniofacial Sciences King's College London London UK; ^2^ Guy's & St Thomas NHS Foundation Trust London UK; ^3^ Private Practice London UK; ^4^ Universitat Internacional de Catalunya Barcelona Spain; ^5^ Endodontic Division, Department of Restorative Dentistry, Faculty of Dental Medicine Umm Al‐Qura University Makkah Saudi Arabia; ^6^ Department of Oral Health Sciences, Endodontology, University Hospitals Leuven KU Leuven Leuven Belgium; ^7^ Boston University Henry M. Goldman School of Dental Medicine Boston Massachusetts USA; ^8^ College of Dentistry University of Baghdad Baghdad Iraq

**Keywords:** external cervical resorption, predisposing factors

## Abstract

**Aim:**

To assess the occurrence of external cervical resorption (ECR) in relation to patient characteristics and potential predisposing factors.

**Methodology:**

In total, 215 ECR lesions (194 patients) referred to the Endodontic postgraduate Unit at King's College London or Specialist Endodontic practice (London, UK) between September 2017 and January 2022. The clinical records were readily accessible for evaluation. At the time of initial presentation, patients diagnosed with ECR were interviewed regarding potential predisposing factors using a checklist based on existing literature of reported predisposing factors. Absolute and relative frequencies for all variables were computed. Inferential analysis was carried out to determine if there was any potential association between the type and location of tooth in the jaw as well as sex, age of the patient and potential predisposing factors.

**Results:**

No identifiable predisposing factor were detected in 22.3% (48/215 teeth) of the cases, 57.7% (124/215 teeth) had a sole, identifiable predisposing factor and 20% (43/215 teeth) had combined (several) factors. The most common (sole or combined) potential predisposing factors were previous/existing history of orthodontic treatment (25.6%, 55/215 teeth), dental trauma injury (DTI) (20.9%, 45/215 teeth), domestic cat ownership (15.8%, 34/215 teeth) and parafunctional habits (10.2%, 22/215 teeth). The highest occurrence of sole predisposing factors was recorded for males (62%, 75/121 teeth), all other ethnic groups combined (non‐white) patients (58.5%, 24/41 teeth), incisor teeth (64.4%, 56/87 teeth) and maxillary teeth (62.4%, 68/109 teeth). While the highest occurrence of combined predisposing factors was found in females (22.3%, 21/94 teeth), white patients (20.1%, 35/174 teeth), premolars (29%, 9/31 teeth) and mandibular teeth (21.7%, 23/106 teeth). There were significant associations between tooth type and trauma (*p* < .001), cat ownership (*p* = .003) and parafunctional habits (*p* = .017). The association between trauma (*p* < .001), cat ownership (*p* = .002) and jaw location were found to be significant. Concerning parafunctional habits, female patients had significantly (*p* = .015) more ECR occurrence than male patients.

**Conclusion:**

Most cases had 1 (sole) identified predisposing factor; orthodontic treatment, dental trauma history and (previous) cat ownership were the most identified factors. The information may be helpful in diagnosing early stage ECR.

## INTRODUCTION

External cervical resorption (ECR) is the loss of dental hard tissue as a result of odontoclastic action. It usually develops in the cervical region of teeth (Patel et al., 2009a). It is a dynamic process involving the periodontal ligament, dental hard tissues, and in advanced cases, the root canal system (Patel et al., 2023a). ECR is initiated by localized damage and/or disruption of the periodontal ligament and the underlying poorly mineralized (pre‐)cementum exposing the underlying dentine resulting in resorption of the dental hard tissues by odontoclasts (Luso & Luder, 2012; Polimeni et al., 2006).

A retrospective, case–control study determined that the prevalence of ECR among patients who had been referred for endodontic problems to a teaching school was 2.3% (Irinakis et al., 2020). In a retrospective study between three endodontic practices in the USA, the prevalence of ECR increased from 0.46% between 2010 and 2015 to 0.96% between 2016 and 2021. One of the suggested reasons for the increased prevalence over these two time periods was the increased use of cone beam computed tomography (CBCT) (Huang et al., 2023).

The aetiology of ECR is complicated and unclear, several potential predisposing factors have been suggested, and these include orthodontic treatment, dental traumatic injuries, restorative treatment and bruxism (Huang et al., 2023; Mavridou et al., 2017; Patel et al., 2018). Studies have assessed possible systemic causes of ECR with diabetes and anti‐resorptive medication being identified as possible aetiological factors (DeLuca et al., 2023; Irinakis et al., 2020). The first extensive study describing possible aetiological factors of ECR was published by Heithersay who collected clinical data on 257 teeth (222 patients). In this study, it was found that orthodontic treatment occurred as a sole or in combination with other factors in 24.1% and 28.4% of cases, respectively, dental trauma injury (DTI) was a sole or in combination with other factors in 15.1% and 25.7% of cases, respectively, and 16.4% of cases had no identifiable aetiological factors identifiable (Heithersay, 1999a, 1999b). Mavridou et al. (2017) assessed 337 teeth (284 patients) and found that orthodontic treatment (45.7%), DTI (28.5%) and parafunctional habits (23.2%) were the most common possible aetiological factor. Ownership and/or exposure to cats has been suggested as a potential predisposing factor for ECR (DeLuca et al., 2023; von Arx et al., 2009).

The aim of this study were to investigate potential predisposing factors of ECR, thus helping to improve the knowledge of the cause and/or risk factors associated with ECR.

## MATERIALS AND METHODS

This clinical strategy was in accordance with PROBE statement (Nagendrababu et al., 2023). This study was a retrospective observational study of 215 ECR cases which were seen in specialist endodontic practice or in the new patient Endodontic unit at King's College London or Endodontic specialist practice (London, UK) between September 2017 and January 2022. Ethical approval was gained by Guy's and St Thomas' NHS Foundation Trust research and development committee* (08/H0804/79, 28/09/17). A study assessing the clinical and radiographic findings of ECR from this cohort of patients has been published (Patel et al., 2023a).

Prior to the study, an extensive search of the literature was carried out to determine potential predisposing local factors for ECR. A checklist of possible predisposing factors to be assessed in this study was formulated (Table 1) for the clinician to use when discussing the potential predisposing factors of the patient's ECR.

**TABLE 1:** The aetiological factors that have been assessed in this study.

Tooth number (FDI)	
Patient sex	
Patient age	
Patient ethnicity	White (English, Welsh, Scottish, Northern Irish or British, Irish, Gypsy or Irish Traveller, Roma, Any other White background) Asian or Asian British (Indian, Pakistani, Bangladeshi, Chinese, Any other Asian background) Black, Black British, Caribbean or African (Caribbean, African, Any other Black, Black British, or Caribbean background) Mixed or multiple ethnic groups (White & Black, White & Black, White & Asian, Any other Mixed or multiple ethnic background) Other ethnic group (Arab, Any other ethnic group)
	Orthodontic treatment Periodontal treatment Parafunctional habits (including bruxism) Tooth whitening (bleaching) Delayed eruption Bisphosphonate medication Dental trauma injury (DTI) Wind instruments Extraction of neighbouring tooth Interproximal stripping Restorations Cracks Poor oral hygiene Cat ownership Development disorder

**FIGURE 1:**
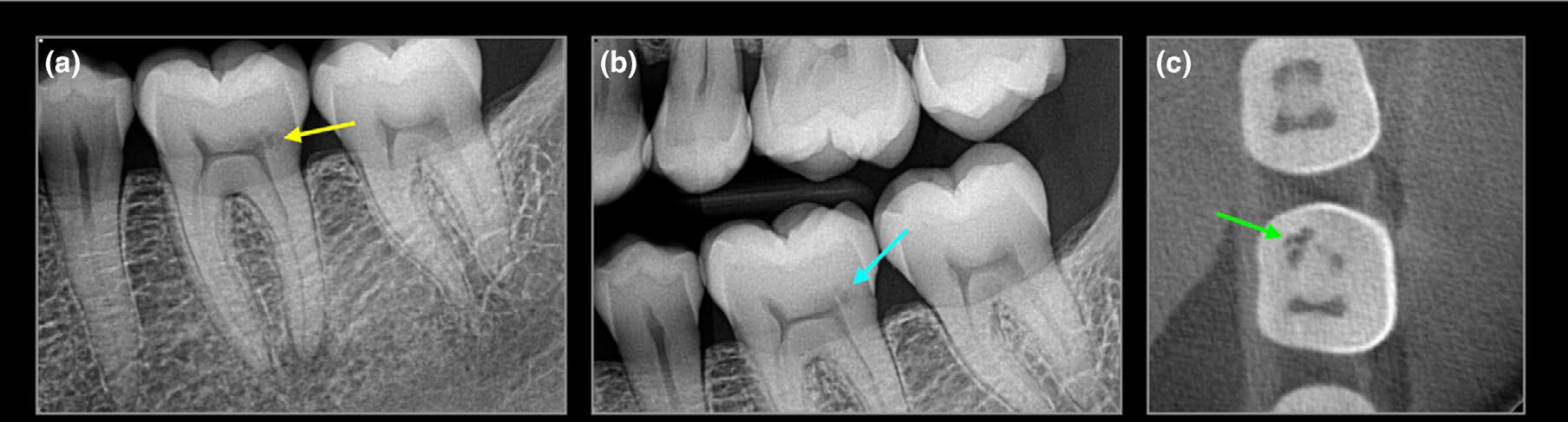
42‐year‐old female with lower left first molar with ECR (a) periapical and (b) bitewing radiograph and (c) axial CBCT slide confirm ECR, potential predisposing factor was parafunction.

**FIGURE 2:**
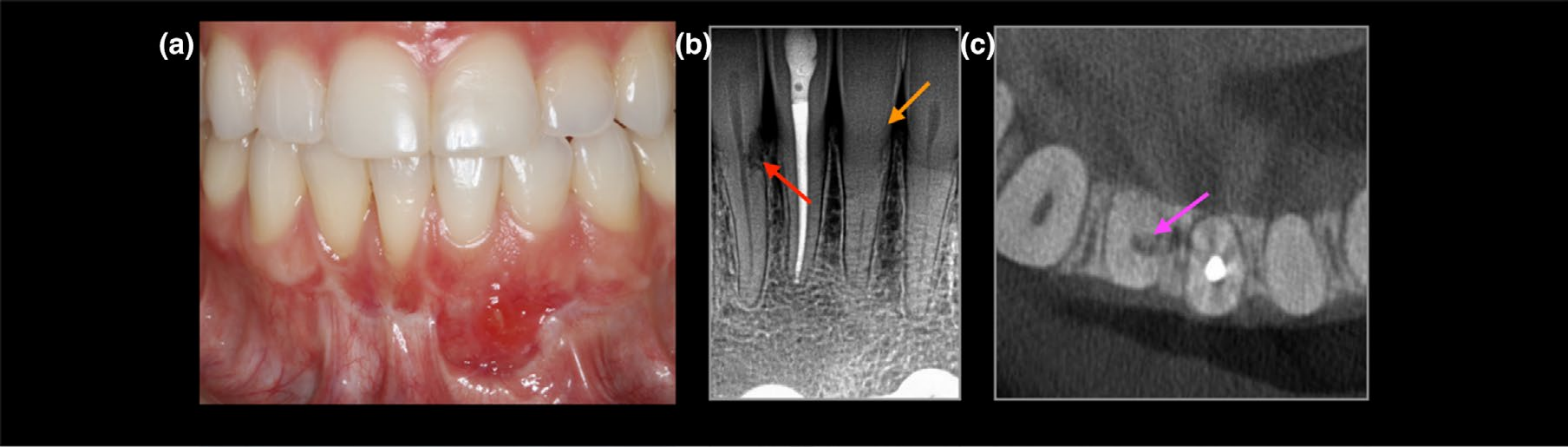
(a–c) 45‐year‐old male with lower right central incisor (a) photo revealing scaring after orthognathic surgery, (b) radiograph reveals ECR in the lower right central incisor (red arrow) and pulp canal obliteration of the lower left central incisor; potential predisposing factors were orthognathic surgery and previous adolescent history of dental trauma.

**FIGURE 3:**
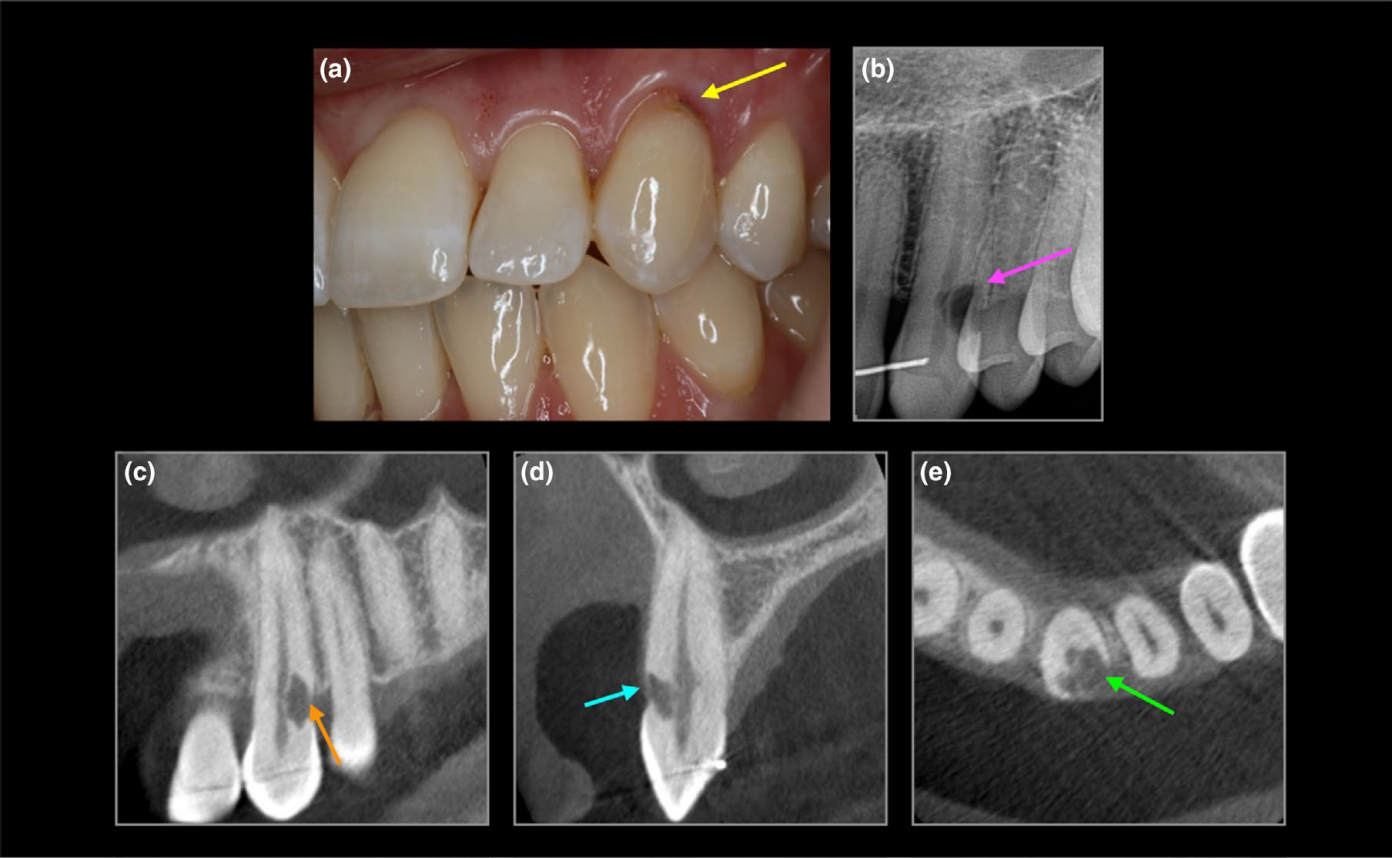
28‐year‐old female with upper left canine with ECR (a) cavitation (yellow arrow) (b) periapical radiograph (purple arrow) (c) coronal CBCT (orange arrow), (d) sagittal (blue arrow) and (e) axial view (green arrow) confirm ECR, potential predisposing factor was dental trauma and cat ownership.

Patients diagnosed with ECR were assessed by an endodontic specialist or a postgraduate endodontic student under the supervision of an endodontic specialist (Figures 1, 2, 3). A proforma of potential predisposing factors was completed at the time of assessment. A detailed medical and dental patient history was taken. A systematic clinical examination which included using a dental operating microscope (6 step entree; Global, St Louis, MO, USA). Any potential predisposing factor was marked on the checklist and stored in the patient notes. A radiographic examination using periapical radiographs (PRs) and CBCT were used to confirm the diagnosis. PR using a beam aiming device with a dental X‐ray machine (Nomad Pro 2 KaVo Dental Ltd., Uxbridge, UK)/digital CCD (Schick Technologies, New York, NY, USA) or Heliodent X ray unit, (Sirona, Bensheim, Germany)/PSP plates using a paralleling technique. The exposure parameters for PR were 60 kV, 7.5 mA and 0.20 s for posterior teeth and 60 kV, 7.5 mA and 0.13 s for anterior teeth. CBCT scans were obtained of areas of interest with 3D Accuitomo scanner (J.Morita, Kyoto, Japan) with 40 × 40 mm field of view and exposure parameters of 90 kV, 5.0 mA and 17.5 s. CBCT scans were reformatted (0.125 slice intervals and 1.5 mm slice thickness).

The data were anonymized and stored in an Excel spreadsheet (Microsoft®, Richmond, WA, USA).

### Data analysis

A sample size calculation was carried out using G*Power software (version 3.1.9.6, Franz Faul, Christian‐Albrechts‐Universität Kiel, Kiel, Germany). For a sample size of 180 patients, a power of 95% would be achieved to detect differences between two independent proportions, assuming a level of confidence of 95%. Descriptive statistics were carried out with SPSS software for Mac (version 28, SPSS Inc., Chicago, IL, USA) to assess data distribution. Absolute and relative frequencies for all variables were computed. Inferential analysis was carried out to determine if there was any potential association between the type and location of tooth in the jaw as well as sex, age of the patient and potential predisposing factors. Chi‐square test was used to compare the association between categorical variables or alternatively, Fisher's exact test to compare distribution among independent groups. The level of statistical significance was set to 5% (*α* = 0.05).

## RESULTS

### Patients' distribution

In total, 194 consecutive patients (215 teeth) diagnosed with ECR were recruited into the study, 88 were female (94 teeth) and 106 were male (121 teeth). The overall mean (± standard deviation) age of patients was 41.5 (±16.5) years, with the mean age of females and males being 42.7 (±17.7) years and 40.4 (±15.4) years, respectively. The age group distribution was: 16–30 years (30.2% [*n* = 65]), 31–45 years (29.8%, [*n* = 64]), 46–60 years (20.9% [*n* = 45]) and 61–81 years (19.1% [*n* = 41]).

### Ethnicity

ECR was diagnosed in 174 White (80.9%) and 41 (19.1%) all other ethnic groups combined [11 Black (5.1%); 20 Asian (9.3%) {8 Asian‐Indian (3.7%); 12 Asian‐Chinese (5.6%)} and 10 Arab (4.7%)].

### Potential predisposing factors

**FIGURE 4:**
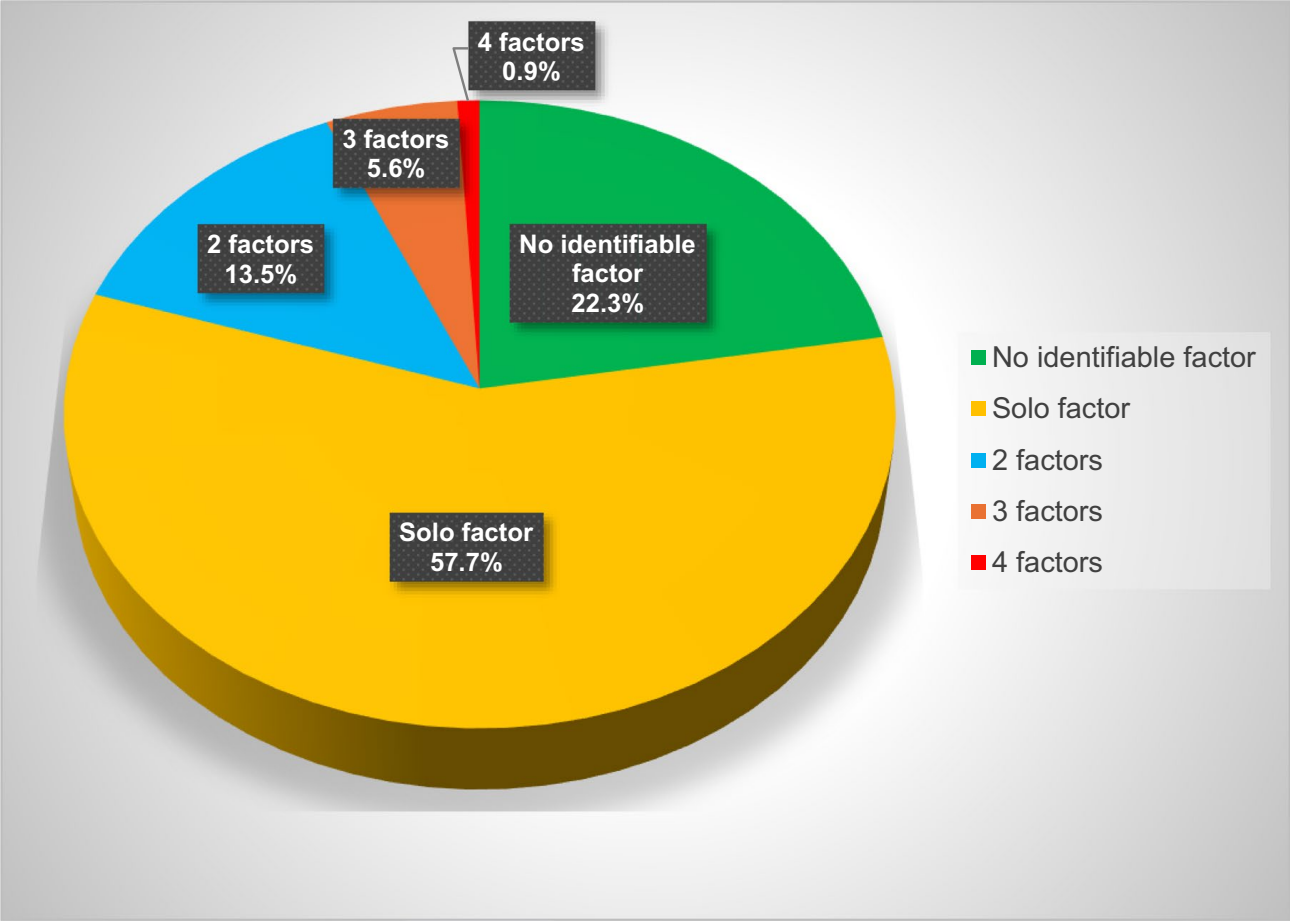
Chart showing the percentage of appearance of the number of factors contributing to the ECR occurrence.

From the examined cases, 22.3% (48/215 teeth) of the cases had no identifiable predisposing factor, 57.7% (124/215 teeth) had a sole, identifiable predisposing factor and 20% (43/215 teeth) has combined (several) factors. The analysis of the number of predisposing factors contributing in the ECR appearance is summarized in Figure 4.

**FIGURE 5:**
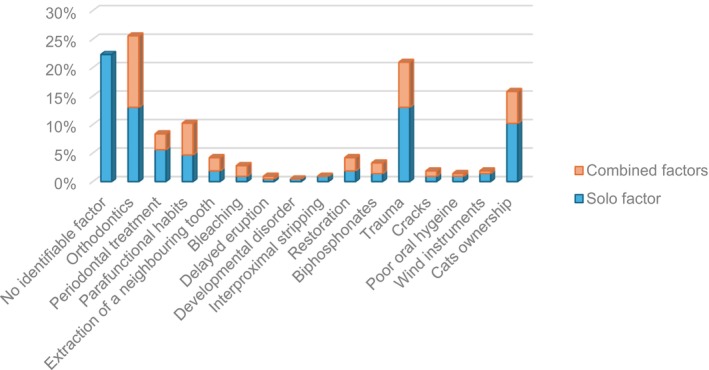
Bar chart showing the percentage of prevalence of ECR as a function of potential predisposing factors as a solo or in a combination with others.

Overall, the most common (sole or combined) potential predisposing factors for ECR were previous/existing history of orthodontic treatment (25.6%, 55/215 teeth), dental trauma (DTI) (20.9%, 45/215 teeth), domestic cat ownership (15.8%, 34/215 teeth) and parafunctional habits (10.2%, 22/215 teeth) (Figure 5).

### Sole versus combined potential predisposing factors

A history of orthodontic treatment was associated as a sole factor to ECR in 13% (28/215 teeth) of cases, and in combination with other potential predisposing factors in a further 12.6% (27/215 teeth) of cases. DTI appeared as a sole factor in 13% (28/215 teeth) of cases and in combination with other potential predisposing factors in a further 7.9% (17/215 teeth) of ECR cases. Domestic pet cats were associated as a sole factor, and in combination with other potential predisposing factors in 10.2% (22/215 teeth) and 5.6% (12/215 teeth) of the cases, respectively. Parafunctional habits were associated as a sole factor and in combination with other potential predisposing factors in 4.7% (10/215 teeth) and 5.6% (12/215 teeth) of cases, respectively (Figure 5).

The frequency of appearance of predisposing factors was significantly linked to the age of the patients (*p* < .001). Sole predisposing factors were most prevalent in (67.7%) of cases in patients aged 30 years old and below, while the highest prevalence of combined factors is observed in patients aged 61 years and above (43.9%) (Table 2).

**TABLE 2:** Frequency of predisposing factors (sole/combination) in relation to the patient's gender, ethnicity, age and tooth type and location in the jaw.

	No identifiable factors % (*n*)	Sole factor % (*n*)	Combined factors % (*n*)	Total % (*n*)
Gender				
Male	19.8 (24)	62 (75)	18.2 (22)	56.3 (121)
Female	25.5 (24)	52.1 (49)	22.3 (21)	43.7 (94)
Ethnicity				
White	22.4 (39)	57.5 (100)	20.1 (35)	80.9 (174)
All other ethnic groups combined	22 (9)	58.5 (24)	19.5 (8)	19.1 (41)
Age (years)				
<30	13.8 (9)	67.7 (44)	18.5 (12)	30.2 (65)
31–45	25 (16)	57.8 (37)	17.2 (11)	29.8 (64)
46–60	28.9 (13)	66.7 (30)	4.4 (2)	20.9 (45)
61+	24.4 (10)	31.7 (13)	43.9 (18)	19.1 (41)
Tooth type				
Incisors	18.4 (16)	64.4 (56)	17.2 (15)	40.5 (87)
Canines	21.2 (7)	51.5 (17)	27.3 (9)	15.3 (33)
Premolars	19.4 (6)	54.9 (16)	29 (9)	14.4 (31)
Molars	29.7 (19)	54.7 (35)	15.6 (10)	29.8 (64)
Jaw location				
Maxilla	19.3 (21)	62.4 (68)	18.3 (20)	50.7 (109)
Mandible	25.5 (27)	52.8 (56)	21.7 (23)	49.3 (106)

The highest occurrence of sole predisposing factors was recorded for males (62%, 75/121 teeth), all other ethnic groups combined (i.e. Black, Asian, etc.) (58.5%, 24/41 teeth), incisor teeth (64.4%, 56/87 teeth) and maxillary teeth (62.4%, 68/109 teeth). While the highest occurrence of combined predisposing factors was found in females (22.3%, 21/94 teeth), white patients (20.1%, 35/174 teeth), premolars (29%, 9/31 teeth) and mandibular teeth (21.7%, 23/106 teeth) (Table 2).

The most frequent combination of potential predisposing factors was orthodontics with a history of DTI (18.6%, 8/43 teeth), orthodontics with parafunctional habits, and orthodontics along with cat ownership (9.3%, 4/43 teeth, each).

### Association between predisposing factors and tooth type

**FIGURE 6:**
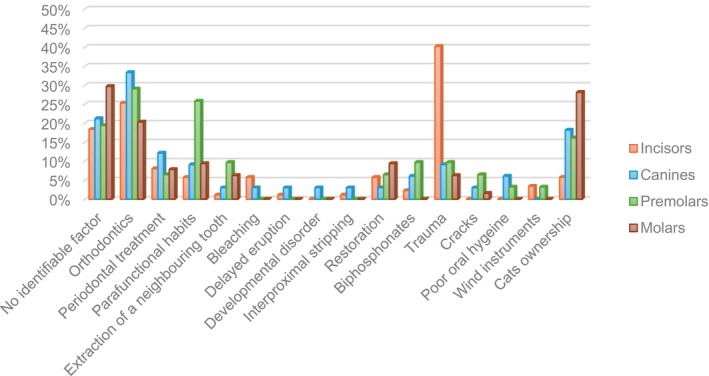
Association between tooth type and predisposing factors.

DTI injuries were associated mostly with incisors (40.2%, 35/87 teeth), and least with molar teeth (6.3%, 4/64 teeth). ECR associated with a history of DTI was significantly more frequent in maxillary incisors (*p* < .001). The most significantly affected teeth associated with cat ownership were molar teeth (28.1%, 18/64 teeth, *p* = .003) (Figure 6).

Parafunctional habits were significantly (*p* = .017) associated with premolars (25.8%, 8/31 teeth), compared to molars (9.4%, 6/64 teeth), canines (9.1%, 3/33 teeth) and incisors (5.7%, 5/87 teeth) (Figure 6).

### Association between predisposing factors and jaw location

**FIGURE 7:**
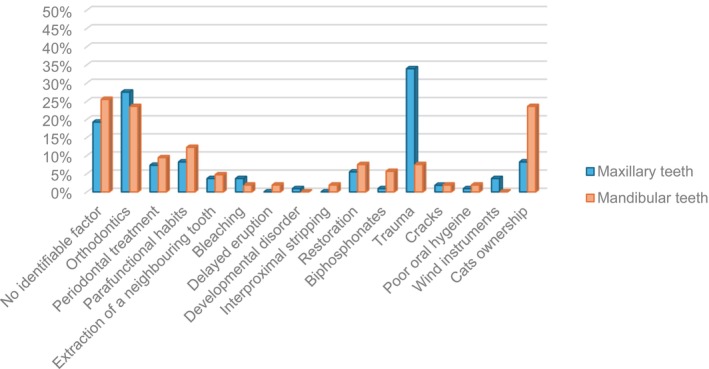
Association between jaw location and predisposing factors.

A history of DTI was significantly associated with ECR in maxillary teeth (*p* < .001) (Figure 7).

### Association between predisposing factors and patient gender

**FIGURE 8:**
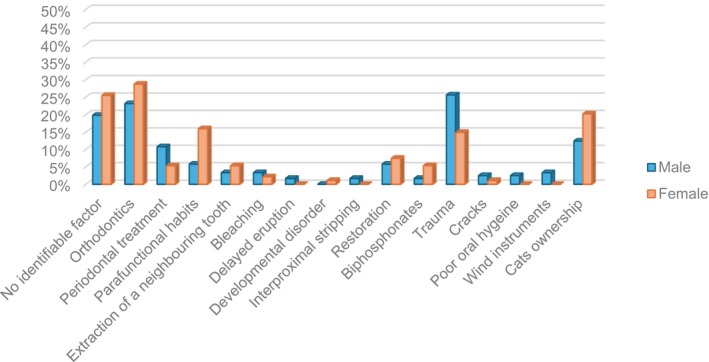
Association between patient gender and predisposing factors.

There was a strong association (*p* = .055) with ECR occurrence caused by a history of dental trauma in male patients (25.6%, 31/121 teeth) compared to female patients (14.9%, 14/94 teeth). There was a significant association between cat ownership and ECR in the mandible (23.6%, 25/106 teeth, *p* = .002) (Figure 8).

Furthermore, cat owners affected by ECR were almost white patients (19%, 33/174 teeth, *p* = .009). Concerning parafunctional habits, female patients (16%, 15/94 teeth) with parafunctional habits had significantly (*p* = .015) more ECR occurrence than male patients (5.8%, 7/121 teeth) (Figure 6).

In cases where orthodontics was identified as a potential predisposing factor, the highest occurrence was recorded for patients less than 30 years old (35.4%, 23/65 teeth). No significant association was observed between the distribution of age and each potential predisposing factor.

## DISCUSSION

It is well established that damage to protective periodontal ligament and underlying cementum can result in resorption (Karring et al., 1984). Several potential predisposing factors have been proposed for the ECR, however, the aetiology of ECR is poorly understood resulting in misdiagnosis and/or inappropriate management (Mavridou et al., 2017; Patel et al., 2018). The aim of this study was to investigate potential predisposing factors associated with ECR as well as to assess prevalence in different ethnic groups.

Large‐scale observational studies on this topic are sparse; to date, the population groups assessed are limited to the USA (DeLuca et al., 2023; Huang et al., 2023) and Belgium (Mavridou et al., 2017). Only 5 and 8 local potential predisposing factors were assessed by De Luca et al. and Haung et al., respectively, only Mavridou et al. (2017) assessed an extensive list of factors, thus reducing the likelihood of missing any potential predisposing factors.

ECR can be challenging to diagnose, and on occasional be misdiagnosed when radiographic assessment is limited to PR (Patel et al., 2009a,b). It is well established that CBCT improves the accuracy of diagnosis of CBCT (Mavridou et al., 2016; Patel et al., 2023a, 2023b). However, the existing studies observational studies assessing predisposing factors published in the last 15 years have not used CBCT for every case resulting in possible under reporting and/or misdiagnosis of ECR. On searching scientific databases, for example, Scopus, PubMed, Web of Science, ERIC, ScienceDirect, Directory of Open Access Journals, the authors could not find any study on the association of ECR with ethnicity and predisposing factors, as well as including a diagnostic CBCT for each case.

The majority of patients presenting with ECR were white (80.9%) which compares to 53.8% of London's demographic being white (Office for National Statistics, 2022). Conversely, 13.5% and 5.1% of patients with ECR were Asian or Black ethnic minorities, respectively, this compared to 20.7% and 13.5% of the greater London population being Asian or Black, respectively (Office for National Statistics, 2022). This difference may be due to disparities in access to dental care in other ethnic minority population groups (Croucher & Sohanpal, 2006). The details of the patient's ethnicity were based on UK 2021 population census classification (https://www.ons.gov.uk/census2021).

The mean age (41.5 years) of diagnosis of patients with ECR in this study was similar (37.9 years) to Mavridou's analysis of 313 teeth with ECR (Mavridou et al., 2022).

A history of orthodontic treatment was found in 25.6% of ECR cases in the present study, a higher prevalence (36.4%–45.7%) was found in other studies (DeLuca et al., 2023; Mavridou et al., 2017). It has been hypothesized that high orthodontic forces in the cervical region of teeth could cause tissue necrosis, which leads to odontoclasts differentiation and resorption of the exposed dentine (Heithersay, 1999a, 1999b). In the present study, the prevalence of a history of orthodontic treatment associated with ECR was significant in patients less than 30 years old. With an increase in orthodontic treatment uptake in adults, an increase in the prevalence of ECR in older patient groups may occur, more research is needed in this area. Initially, patients who recalled having orthodontic treatment were asked about the type (e.g. fixed, removal, aligners), nature and duration of orthodontic treatment, however, it became evident that the majority of patients could not recall the specific nature of their treatment as in some cases the treatment may have been carried out 40+ years ago (age range for patients with orthodontic treatment is 17–74 years). Future studies should also assess whether there are any differences in ECR association with fixed braced, particularly the type (for example, labial, lingual, expansion, headgear, functional), as well as removable appliance including clear aligners as well as the duration of treatment.

In the present study, 20.9% of patients with ECR had a history of DTI, other studies have found a prevalence of DTI to be 25.7%–32.2% (DeLuca et al., 2023; Heithersay, 1999a, 1999b; Mavridou et al., 2017). The high prevalence of TDI in school children (25%) and adults (33%) would help explain relatively high proportion of patients with TDI deemed to be a potential aetiological factor of ECR (Glendor, 2008). This would also explain the correlation of TDI as a predisposing factor and ECR on maxillary incisors as well as the higher prevalence in males compared to females, similar results were reported by Mavridou et al. (2017).

von Arx et al. (2009) hypothesized the possible transmission of a feline virus to humans (i.e. domestic cats to their owners) as a cause of ECR. It has been reported that this virus may induce osteoclastogensis (Boyle et al., 2003). The prevalence of ECR (feline osteoclastic resorptive lesions) in cats, ranges from 28.5% to 67.0%, and the incidence increased with age (Gorrel, 2015; Lommer & Verstraete, 2000). It has been reported that between 22% and 23% of the population own a cat in England (Murray et al., 2015; Westgarth et al., 2010), therefore, the reported prevalence (25.5%) of ECR in the present study appears to be plausible, if indeed there is a transfer of virus from cats to their owner. DeLuca et al. (2023) found that 13.3% of patients they diagnosed with ECR owned cats. In the current study, ECR affected premolars and molars rather than incisors when cat ownership was identified as a potential aetiological factor. However, it should be noted that the patients who had or did own a cat were not tested for feline herpes virus type 1 antibodies. Future studies should consider the potential role of other viral infections, for example, herpes simplex, herpes zoster, cytomegalovirus in ECR (Ramchandani & Mellor, 2007; Solomon et al., 1986).

Reduced vascular perfusion cervically as a result of parafunction may lead to changes in the oxygen tension resulting in a short‐term, hypoxic microenvironment in the cervical region which can result in a two to fourfold increase in osteoclastic activity which may result in ECR of the damaged root surface (Mavridou et al., 2019). An umbrella review determined the prevalence of awake and sleep bruxism was 30% and 15%, respectively (Melo et al., 2019), therefore, the portion of patients (10.2%) with ECR who had a history and/or signs of parafunction (bruxism) was not surprising. ECR in teeth attributed with a bruxism (parafunction) aetiology were predominately posterior teeth [premolar (26.3%) and molar (12.2%) teeth], which would be consistent with these tooth groups being most frequently affected with eccentric loading in lateral excursions, leading to a potential overload and chronic inflammation of the periodontal ligament (Chen et al., 2021; Wood et al., 2008).

In the present study, only 2.8% of teeth with ECR were associated with intracoronal bleaching, and this was in the same order of magnitude (<3%) as other studies (DeLuca et al., 2023; Mavridou et al., 2017). A previous study found that 13.6% of cases had a history of intracoronal bleaching (Heithersay, 1999a, 1999b) which may be attributed to heat application and/or high‐concentration bleach being applied. The lower prevalence in the present study may be in part due to the moving away from using heat to accelerate the bleaching process as well as only lower concentration bleach being used in the UK (General Dental Council, 2016; Harrington & Natkin, 1979; Malik, 2018).

It is possible that systemic illnesses and/or associated medication, particularly those that influence bone metabolism and turnover may be associated with ECR (Huang et al., 2023). Furthermore, Bisphosphonates (BSP), have been implicated in causing ECR (Patel & Saberi, 2015), and it has been reported that Alendronic acid indirectly causes ECR by releasing proinflammatory cytokines resulting in resorption. Paradoxically, discontinuation of Denosumab has been associated with increased prevalence of ECR (Alyahya & Myers, 2021); more research is needed in this area.

Over the last four decades, several local risk factors have been reported to be associated with and/or suggested as potential predisposing factors for ECR. Previous studies have assessed systemic factors such as diabetes and hypertension as potential predisposing factors for ECR (Huang et al., 2023; Irinakis et al., 2020). The present study focused on local factors as a specific link between systemic illnesses and ECR have not been established. Further biological and immunological studies are required as they may yield more information whether there is any association or causation between ECR and systemic health (Jeng et al., 2020).

Despite a checklist of potential predisposing factors being used, no causes were identified in a significant number of the patients (22.3%), this compares to previous studies finding 16.5% (Heithersay, 1999a, 1999b) or, even less, 1% (Mavridou et al., 2017) of cases as idiopathic. This may be due to recollection bias, that is, patients underreporting their past history of trauma, parafunctional habits and/or any other non‐evident factor (Frissa et al., 2016; Krayem et al., 2021). It may also be due to ECR being idiopathic in nature in some cases.

In this study, poor oral health was less prevalent (1.5%) as a sole associated factor, compared 5% of cases identified by of Mavridou et al. (2017). This may be due to the need of primary dental disease stabilization to access the secondary (specialist) care offered by a National Health Service in the UK. Patients seen in private practice usually tend to be more motivated to maintain their oral hygiene as well as see the hygienist regularly. The oral health assessment screen was used to provide an objective means of assessing oral health (www.nice.org.uk).

## CONCLUSION

The main potential predisposing factors associated with the ECR presence were a history of orthodontic treatment (25.6%), TDI (20.9%), cats (15.8%) and parafunctional habits (10.2%). A significant portion of patients had no identifiable predisposing factor. More large‐scale, prospective studies are needed to identify risk factors.

## AUTHOR CONTRIBUTIONS

S. Patel: conceptualization, methodology, visualization, resources, writing – original draft, writing – review & editing, project administration. Francesc Abella, Kreena Patel, Nassr Al‐Nuaimi: writing, resources, review & editing. P Lambrechts: writing – review & editing.

## CONFLICT OF INTEREST STATEMENT

The authors deny any conflicts of interest.

## Data Availability

Data sharing is not applicable to this article as no new data were created or analyzed in this study.
